# Standard median sternotomy, right minithoracotomy, totally thoracoscopic surgery, percutaneous closure, and transthoracic closure for atrial septal defects in children: A protocol for a network meta-analysis

**DOI:** 10.1097/MD.0000000000017270

**Published:** 2019-09-20

**Authors:** Kang Yi, Xujian Guo, Tao You, Yunfang Wang, Fan Ding, Xiaodong Hou, Li Zhou

**Affiliations:** a Department of Cardiovascular Surgery, Gansu Provincial Hospital; b International Congenital Heart Disease Diagnosis and Treatment Reginal Center; c The First Clinical Medical College of Lanzhou University; d Department of Endocrinology, Gansu Provincial Hospital; e Gansu Provincial Maternity and Child-care Hospital, Lanzhou, China.

**Keywords:** atrial septal defect, child, network meta-analysis, percutaneous closure, right minithoracotomy, standard median sternotomy, thoracoscopic surgery, transthoracic closure

## Abstract

**Background:**

Atrial septal defect (ASD) is one of the most common congenital heart diseases, with an average of 1.64 per 1000 newborns with the ASD. Empirical studies suggest that surgery should be performed early in the presence of right atrium and or right ventricular enlargement, even for asymptomatic patients. Many surgical procedures can be used to treat ASD. But which method is the best choice remains unclear. This study aims to compare the efficacy and safety of standard median sternotomy, right minithoracotomy, totally thoracoscopic surgery, percutaneous closure, transcutaneous by echocardiography, and transcutaneous by radiotherapy for ASDs in children using Bayesian network meta-analysis (NMA).

**Methods:**

We will perform a comprehensive literature search using PubMed, EMBASE.com, the Cochrane Library, Web of Science, and Chinese Biomedical Literature Database to identify relevant studies from inception to April 2019. Randomized controlled trials, prospective or retrospective cohort studies that reported the efficacy and safety of surgical procedures for the treatment of atrial septal defects will be included. Risk of bias of the included randomized controlled trials and prospective or retrospective cohort studies will be evaluated according to the Cochrane Handbook 5.1.0 and the risk of bias in non-randomized studies of interventions, respectively. A Bayesian NMA will be performed using R 3.4.1.

**Results:**

The results of this NMA will be submitted to a peer-reviewed journal for publication.

**Conclusion:**

This NMA will summarize the direct and indirect evidence to assess the efficacy and safety of different surgical procedures for the treatment of ASDs.

**Ethics and dissemination:**

Ethics approval and patient consent are not required as this study is a network meta-analysis based on published trials.

**PROSPERO registration number:**

CRD42019130902.

## 1 Introduction

Congenital heart disease (CHD) is the most common congenital malformation in birth defects and is the main cause of neonatal death.^[1]^ Its treatment has become an important public health problem today and has received extensive attention.^[1]^ Atrial septal defect (ASD) is one of the most common congenital heart diseases, accounting for about 13% of all CHD, with an average of 1.64 per 1000 newborns.^[2]^ ASD may initially be asymptomatic, but empirical studies suggest that surgery should be performed early in the presence of right atrium and/or right ventricular enlargement, even for asymptomatic patients.^[3]^

Many surgical procedures can be used to treat atrial septal defects. The median sternotomy under cardiopulmonary bypass, as the gold standard, is the standard method for the treatment of ASD.^[4]^ Although the mortality and morbidity rate of ASD patients treated with median sternotomy is 0%, it can cause greater trauma to patients.^[5]^ The minimal right minithoracotomy is a minimally invasive and aesthetic technique. Compared with the traditional median thoracotomy, this surgical method avoids mediastinal trauma, reduces postoperative drainage and pain, improves postoperative recovery, prevents postoperative pectus carinatum, shortens postoperative hospital stay, reduces hospitalization costs, and has cosmetic effects. In recent years, it has been widely used.^[5–7]^ In 1976, King et al first proposed percutaneous intervention with an occlusion device to treat ASD.^[8]^ Percutaneous interventional closure of ASD usually requires joint monitoring and guidance of X-ray and transthoracic echocardiography, which has the characteristics of small trauma, rapid recovery, and no extracorporeal circulation.^[9,10]^ In 2000, Xijing Hospital in China combined the catheter sealing technique with the right minithoracotomy to complete the transthoracic ASD closure for the first time.^[11]^ This procedure involves a 2 to 3 cm incision in the right chest wall, which is guided under ultrasound and does not require extracorporeal circulation and fluoroscopy.^[11–13]^ With the continuous deepening and promotion of minimally invasive concepts and techniques in cardiac surgery, in recent years, the application of complete thoracoscopic techniques in the repair of atrial septal defect has also increased.^[14,15]^

Recently, some pairwise meta-analyses have compared the efficacy and acceptability of some procedures.^[16–19]^ But which method is the best choice remains unclear. Network meta-analysis (NMA) can estimate the relative effectiveness of all interventions and the sequence of interventions, even in the absence of a head-to-head comparison of all interested interventions.^[20–22]^ Thus, our study aims to compare the efficacy and safety of standard median sternotomy, right minithoracotomy, totally thoracoscopic surgery, percutaneous closure, and transthoracic closure for atrial septal defects in children using Bayesian network meta-analysis.

## 2 Methods

This protocol will be reported according to preferred reporting items for systematic review and meta-analysis protocols (PRISMA-P),^[23]^ and this network meta-analysis will be performed and reported in accordance with PRISMA extension version (PRISMA-NMA).^[24]^ This study protocol has been registered on the international prospective register of systematic review (PROSPERO) (CRD42019130902).

### 2.1 Search strategy

We will perform a comprehensive literature search using PubMed, EMBASE.com, the Cochrane Library, Web of Science, and Chinese Biomedical Literature Database to identify relevant studies from inception to April 2019. The reference lists of published reviews and included articles will be checked for additional trials. The PubMed search strategies as follows:

#1 “Heart Septal Defects, Atrial”[Mesh]
#2 Atrial Septal Defect∗[Title/Abstract] OR ASD[Title/Abstract]
#3 #1 OR #2
#4 “Minimally Invasive Surgical Procedures”[Mesh] OR “Thoracoscopy”[Mesh]
#5 sternotomy[Title/Abstract] OR “minimally invasive”[Title/Abstract] OR “Minimal Access Surgical Procedures”[Title/Abstract] OR mini-invasive[Title/Abstract] OR “Minimal Surgical Procedure∗”[Title/Abstract] OR “surgical closure”[Title/Abstract] OR “percutaneous occlusion”[Title/Abstract] OR transcatheter[Title/Abstract] OR thoracoscop∗[Title/Abstract]
#6 #4 OR #5
#7 #3 AND #6

### 2.2 Eligibility criteria

#### 2.2.1 Types of study

We will include randomized controlled trials, prospective or retrospective cohort studies that reported the efficacy and safety of one of the 6 methods for the treatment of atrial septal defects. The number of cases included in each group must be greater than 10. Relevant systematic reviews or meta-analyses will be also included to track their references.

#### 2.2.2 Participants

Children younger than 18 years of age with atrial septal defects confirmed by clinical and transthoracic echocardiographic and scheduled for standard median sternotomy, right minithoracotomy, totally thoracoscopic surgery, percutaneous closure, and transthoracic closure repair.

#### 2.2.3 Interventions

Standard median sternotomy, right minithoracotomy, totally thoracoscopic surgery, percutaneous closure, transcutaneous by echocardiography, transcutaneous by radiotherapy, or one of these 6 methods combines with another one.

#### 2.2.4 Outcomes

The primary outcomes will include surgical success rate, operation time, total postoperative complication rate, postoperative major complication rate, any residual shunt after procedure, incidence of arrhythmia, and incidence of pericardial effusion. The secondary outcomes are total hospital stay, postoperative hospital stay, and total cost.

#### 2.2.5 Other criteria

For similar studies published by the same author or institution, an article with a long follow-up or a larger number of studies will be included. The exclusion criteria are

1. the study group contained other congenital heart diseases (such as ventricular septal defect and patent ductus arteriosus);
2. comparison of cardiac function and cardiac physiology changes after atrial septal defect treatment;
3. narrative review or conference abstract.

### 2.3 Selection of studies

We will import the literature search records into EndNote X8 (Thomson Reuters (Scientific) LLC Philadelphia, PA, US) software. The title and abstract of studies found in the search will be examined by 2 independent reviewers to identify related studies according to eligibility criteria. Then, the same 2 reviewers will explore the full-text versions of all potentially relevant studies. Excluded trials and the reasons for their exclusion will be listed and examined by a third reviewer.

### 2.4 Data extraction

We will create a data abstraction form using Microsoft Excel 2013 (Microsoft, Redmond, WA) to collect data of interest. Then, 2 independent reviewers will extract the basic characteristics and the data of outcomes of the included studies. The extracted data will include: first author, publication year, country, study design, study time, arms, sample, mean age, mean body weight, gender, type of surgery, method of surgical closure, device used, median follow-up, and outcomes. If there is a discrepancy between the two reviewers, a third researcher will be consulted.

### 2.5 Risk of bias assessment

We will use the tool of risk of bias in non-randomized studies of interventions (ROBINS-I)^[25]^ to assess the risk of bias of the included prospective or retrospective cohort studies. The risk of bias will be ranked as low, moderate, serious, critical risk of bias and no information. If random controlled trials are included, we will use the Cochrane Handbook version 5.1.0 to assess its risk of bias, and we will evaluate the risk of bias as low, high or unclear. The risk of bias assessment will be completed by two independent reviewers, and conflicts will be resolved by a third reviewer.

### 2.6 Geometry of the network

We will create a network plot to describe and present the geometry of the treatment network of comparisons across trials using STATA (13.0; Stata Corporation, College Station, TX). If the trial is not linked by treatments, we will exclude it from network meta-analysis and just describe the findings of the study. In the network plot, nodes represent different interventions and edges represent a head-to-head comparison between interventions. The size of the nodes and the thickness of the edges are associated with the sample size of the intervention and the number of trials included, respectively.

### 2.7 Statistical analysis and data synthesis

#### 2.7.1 Pairwise meta-analysis

Pooled odds ratio (OR) with 95% credible intervals (CrIs) will be used for the dichotomous variable. Weighted mean differences or standardized mean differences with 95% CrI will be used for the continuous variable. Conventional meta-analyses will be conducted using a random-effects model. The heterogeneity between head-to-head trials will be estimated using I^2^ statistics. The values of 25%, 50%, and 75% for the I^2^ as indicative of low, moderate, and high statistical heterogeneity, respectively. We will explore sources of heterogeneity by subgroup analysis and meta-regression. If there is no clinical heterogeneity, the random-effects model will be used to perform the meta-analysis. Otherwise, clinical heterogeneity will be explored through discussion with the review team.

#### 2.7.2 Network meta-analysis

NMA will be conducted on both direct evidence and indirect evidence in a Bayesian framework using a random-effect model or a fixed-effect model. We will use the deviance information criterion (DIC) to compare model fit and parsimony. The convergence will be assessed using the Brooks-Gelman-Rubin (BGR) plots method. The node splitting method will be used to examine the inconsistency between direct and indirect comparisons if a loop connecting 3 or more arms exist.^[26]^ If node-splitting analysis determined *P* < .05, the inconsistency model will be used for pooled analysis. Otherwise, the consistency model will be used.^[27,28]^ Besides, rank probabilities will be calculated, which indicate the probability for each treatment to be best, second best and so on. The analyses will be performed using R (version 3.4.1; R Foundation for Statistical Computing, Vienna, Austria) software.

#### 2.7.3 Subgroup analysis

If the necessary data are available, subgroup analyses will be done for different types of participants by age, gender, and country.

### 2.8 Assessment of publication bias

The funnel plot and Egger test will be conducted to detect publication bias if the number of studies more than 10.

### 2.9 Quality of evidence

We will assess the quality of the evidence using the Grading of Recommendations Assessment, Development and Evaluation (GRADE) approach to assess the quality of the body of evidence. The GRADE approach uses five considerations (study limitations, consistency of effect, imprecision, indirectness and publication bias) to assess the quality of the body of evidence for each outcome. It is classified into 4 levels: high level, moderate level, low level, and very low level.^[29]^

## 3 Ethics and dissemination

Ethical approvals and patient consent are not required because this is a network meta-analysis based on published trials. The findings of this project will provide a general review and evidence of the efficacy and safety of standard median sternotomy, right minithoracotomy, totally thoracoscopic surgery, percutaneous closure, transcutaneous by echocardiography, and transcutaneous by radiotherapy for the treatment of atrial septal defects in children. The results will be submitted to a peer-reviewed journal for publication. We hope that these findings will help clinicians and patients choose a more appropriate repair method for atrial septal defects.

## Author contributions

**Conceptualization:** Xujian Guo, Tao You.

**Data curation:** Kang Yi, Xujian Guo, Tao You, Li Zhou.

**Funding acquisition:** Kang Yi.

**Investigation:** Kang Yi, Yunfang Wang, Xiaodong Hou.

**Methodology:** Kang Yi, Tao You.

**Resources:** Xujian Guo, Yunfang Wang, Fan Ding, Xiaodong Hou, Li Zhou.

**Software:** Fan Ding.

**Supervision:** Tao You.

**Validation:** Tao You.

**Visualization:** Yunfang Wang.

**Writing – original draft:** Kang Yi, Xujian Guo, Tao You.

**Writing – review & editing:** Kang Yi, Xujian Guo, Tao You.
